# Mr. Fawdington on Subcutaneous Nævus, with Remarks

**Published:** 1830-10-01

**Authors:** 


### XXXIII.

### Subcutaneous Nævus treated by the Seton. By Mr. Fawdington.*

*Case* I. J. Gaskell, three weeks old, was brought to Mr. Fawdington, in June 1825, with a subcutaneous nævus of a bluish colour, about the size of a walnut, situated deeply behind the angle of the jaw. It had been first observed a fortnight after birth. An astringent lotion was prescribed, but in six weeks the disease had made considerable progress. It had taken deep and firm root between the mastoid process and the angle of the jaw; was of oval shape, and measured from above downwards 5 inches and a quarter, by 4 inches in its transverse direction. Above it extended as far as the zygomatic arch, surrounding the ear, and elevating its lobe; below it passed to three quarters of an inch below the level of the base of the jaw; anteriorly it reached to midway between the angle and the symphysis; and posteriorly its most prominent part would project a little more than an inch beyond a perpendicular line drawn through the meatus auditorius externus. It was soft and compressible, possessed a slight thrill, was of purplish colour, and large veins ran conspicuously over it. In various parts, especially just beneath the ear, the capillaries of the skin were assuming the state of the cutaneous nævus. The cries of the child, or any similar exertion, augmented the size of the tumour; the general health continued unimpaired.

Mr. Fawdington, reasoning on erroneous premises, determined to tie the carotid artery, which he did with some difficulty on the 9th of July. The ligature separated from the vessel on the 17th, but, as might be readily anticipated, the tumour was little diminished by the operation. On the 24th, an undulatory motion, not amounting to pulsation, was distinguished in the temporal artery. On the 20th of August all hopes of success from the ligature of the carotid artery were abandoned, and Mr. F. made trial of the method recommended by Mr. Abernethy — pressure and cold applications. At the end of a fortnight the surface was excoriated, and the size of the tumour increased.

On the 10th September a seton was passed through the tumour in its long direction. Precaution was taken to make the skein of sufficient size to close and compress the apertures made by the needle in order to obviate the danger of hæmorrhage. A kind of boil formed at the upper part of the tumour, which was partially inflamed, and appeared to become the seat of several small abscesses opening into the track of the seton. On the 26th, no traces of the morbid structure were visible in the upper two-thirds of what had been the tumour, except that the surrounding veins continued larger than usual. The lower third had, however, undergone no change, and a seton was therefore passed transversely through it. As in the former instance, inflammation, suppuration, and destruction of a great part of the tumour ensued; but one part, the size of an almond, remaining, the seton was withdrawn, and the nitrate of silver introduced through a canula passed in its track, was applied to this stubborn remnant of the malady. On the 2d of February not a

* North of England Med. Journ. No. I.

trace of the morbid growth remained. The cicatrices were insignificant, but accompanied with some degree of puckering and depression of the skin. On taking a front view of the face a slight irregularity was observable in the contour of the side affected, and it likewise appeared rather fuller than the opposite.

The case is accompanied with two coloured lithographic plates, exhibiting the condition of the patient before and after the adoption of the measures we have mentioned. The following are Mr. Fawdington's comments on the subject.

"The ligature of the carotid in the foregoing case arrested the increase of the swelling, but appears altogether to have failed in its cure. This circumstance would excite no surprise, when we regard the numerous vascular inosculations existing in the situation of the tumour, if there were not cases on record of indisputable success from the operation in question. Besides those related in the Medico-Chirurgical Transactions by Mr. Dalrymple and Mr. Travers, which, by the way, appear to have been instances of arterial nævus, and one subsequently by Mr. Wardrop, clearly the venous nævus, in which the utility of the operation was more questionable, the latter gentleman has recently had the most complete success from tying the carotid in a case, so far as I can perceive, very similar to the one which is the subject of these remarks. The cause of failure, then, in the present example, I shall leave it to the profession to solve ; and, while I am inclined to entertain a preference for the seton in similar cases, as regards situation and extent, I take leave to say that, it is only in consequence of the experience developed in this paper, on the one hand, favourable to the latter measure, and, on the other, discouraging in respect to the former.* Both measures, I am aware, required further trial ; and it was the disappointment in the result of tying the artery, and an unexpected degree of success from the process by the seton in this case, which induced me, with Dr. Hull's concurrence, to adopt the latter in the following instance ; a choice between the two, in consequence of the size and situation of the tumour, being the only advantage left to me."

We are not acquainted with Mr. Wardrop's successful case. If Mr. Fawdington alludes to the one that occurred at Panton Square, he is mightily mistaken in his ideas of the result. Has Mr. F. been blinded by the impudent mendacity of the lying Journal ? Is he ignorant of the base metal it palms upon its customers as current coin ? The bare appearance of a case in that magazine of mendacity, is sufficient to involve it in doubt and suspicion. With respect to Mr. Travers' case, it is generally acknowledged that the cure was not wholly attributable to the operation. But allowing that one or two such tumours have been benefited by tying the carotid, the instances of failure have far out-numbered those of success. Such an operation is not a bagatelle, nor a step to be tried in the way of an experiment. In the case of Dr. Macfarlane, of which we have given an account, the patient died in consequence of its performance, and in that of Mr. Wardrop, he also sank from its remote effects. Such warnings should not be thrown away, nor are we rashly and obstinately to expose individuals to destruction. We have, on former occasions, protested against the application of the Hunterian operation for aneurism to affections of this description. The best

* "The uncertain result of this operation is well exemplified in the communication of Dr. Mussey, which may be read in the Number of the London Medical Gazette for April 17th, 1830. In this case, the tumour was situated on the vertex, and *both* carotids were tied within twelve days, with little permanent advantage, the disease afterwards requiring to be extirpated. This was done six weeks after tying the second artery, at the expense of a considerable share of hemorrhage ; from the consequences of which, however, the patient eventually recovered."

informed members of the profession are of the same opinion, and it behoves less experienced men to be chary in their attempts to gain operative fame.

*Case* 2. Eliz. Tetlow, ten months old, had a small subcutaneous nævus on the forehead since birth, which, during the last two months, had increased so as to occupy the left half of the forehead, encroaching on the eyelid, and extending over the anterior half of the corresponding parietal bone. It presented the venous character, was of leaden colour, and had no pulsation. The cutis did not participate in the disease, and although the eye-lid could not be voluntarily raised, yet on raising the palpebra, no trace of it could be discerned beneath the conjunctiva.

The seton was passed through the long diameter of the tumour and retained for nearly three weeks, but the diseased part was only destroyed in the immediate neighbourhood of the thread. It was therefore withdrawn, and a strong solution of the sulphate of copper injected into the channel left by it. A degree of inflammation succeeded, but the lateral parts of the nævus remained unaltered. Another seton was introduced transversely with partial benefit only, and a stick of the nitrate of silver was introduced into the opening. Extensive and severe inflammation followed and threatened to run into sloughing, but this was avoided by soothing applications. Chronic inflammation was left behind, and occasional leeching with evaporating washes were required. At the end of three months every trace of the disease had disappeared, but the inflammation occasioned by the nitrate of silver had spread to the palpebral conjunctiva, and occasioned granulations and thickening. The granulations were excised, yet the eyelid did not admit of being completely closed, and the veins in the vicinity remained more dilated than natural. Some, but not much, disfiguration remains, whilst the eye-lid still continues thickened and incapable of being elevated fully.

"Though the seton in this case was not alone competent to cure the nævus, yet, it must be confessed, that it contributed in a principal degree to that end, and afforded the opportunity, additionally, of modifying the application of caustic in such a manner as to preserve the integument. It is obvious from the situation and dimensions of the tumour that neither ligature, excision, nor the caustic, in the usual way of employing it, could have been judiciously adopted; for independently of the risk of hemorrhage, or the deformity which would have been thus occasioned, the particular functions of the parts implicated, especially the eye-lid, would have been essentially impaired. Though the progress of the cure was tedious, and the little patient at one time apparently in hazard from excessive inflammation of the part, the result, upon the whole, was gratifying; and the case instructive, as it teaches the fact, that, caustic applied internally to the morbid growth, does not necessarily involve the destruction of its integuments; at the same time, that we should be moderate in its use when thus employed: for, it is to be remembered, that this agent is not intended to act so much in directly disorganizing the diseased texture, as in setting up a destructive inflammation, by which its obliteration appears to be effected. Upon this principle, indeed, the method of treatment by seton is recommended."

*Case* 3. George Crowther, a healthy child, ten months old, affected with a nævus, chiefly subcutaneous, yet partly affecting the cutis, about the size of the section of a pullet's egg, projecting from the forehead immediately above the left eye-brow. It began as a small red cutaneous spot, when the child was three weeks old. On the 7th April, 1828, a seton was passed through the tumour. Some swelling and inflammation followed; and on the 20th the seton was removed. On the 1st of May the tumour was about half its former size; on the 7th another seton was introduced transversely. This caused greater inflammation and swelling than the former, and the whole of the nævus appeared to be obliterated. On the 19th this seton was taken out likewise.

Much induration of the parts included within the seton openings remained behind, and after awhile a portion of nævus the size of a horse-bean re-appeared. It is proposed to remove this by excision.

"We are therefore entitled to conclude, that though the seton has not completely succeeded in this instance, in obliterating the morbid structure, it has arrested its growth, and reduced it within a compass, which allows of the interposition of another remedial agent that might be applied safely, and insure the least imaginable deformity. I am quite aware that either excision or the ligature was applicable in the first instance; the latter especially, might have been adopted with certainty, and even the former would not have been attended with hazard, if a competent assistant had been employed to compress steadily the circumference of the incision, while the operation was in progress. But with the view of creating as little disfigurement as possible, I was led, from the evidence of the preceding cases, to select the seton; and the event, though not such as to entitle it to be called 'a perfect cure,' appears to warrant the procedure, and to be not altogether unworthy of the notice of the practical surgeon."

Such are the cases adduced by Mr. Fawdington in advocacy of the employment of the seton for nævi materni. Our readers may form their own conclusions on its powers and advantages. For our parts we see but little to recommend it in preference to the means in common use. If a surgeon is consulted in the early stages of the tumour, he certainly has no business to allow it to attain considerable magnitude, and, as far as we have seen, we should say that the disease is seldom allowed to advance very far before his assistance is required. For moderate sized nævi then, we have no hesitation in asserting that excision or the ligature is infinitely preferable to the seton; and the ligature may be employed though the tumour should have attained a considerable size. The case lately published by Mr. Brodie is sufficient to establish the truth of this position.

It is obvious, however, that the nævus may be of such magnitude, or so situated in such a particular spot, as to render excision or the ligature improper. In such cases our readers are put in possession of the experience of Mr. Fawdington, respecting the value of the seton. Let us glance at the results. In the first case the seton was required to be twice introduced, and at last the nitrate of silver was necessary; abscesses and sloughs formed in the nævus, and a good deal of constitutional irritation was induced; the cure was complete. In the second, the seton was twice introduced, sulphate of copper and nitrate of silver were applied, severe disturbance followed the latter, and the nævus was only cured, with granular and thickened palpebral conjunctiva. In the third, the seton was also passed twice, but a portion of nævus remained for excision. This is the true state of the question, and practitioners may see that although the seton may prove useful in *some* cases, it is not to be deemed a very efficient, nor yet a very mild mode of treatment. Such at least may be deduced from the foregoing facts, if so few can be fairly considered as entitled to settle a point of this description. By the way we should mention that Mr. F. takes much pains to draw the line between subcutaneous nævus, appearing to be chiefly a venous structure, and the pulsating nævus of arterial and aneurismal character. He might have spared himself much trouble on this point, as the distinction was drawn long ago. He will find it, no doubt, in Cooper's Surgical Dictionary. On the whole, our author's paper is interesting, and the facts it contains are valuable. If we differ in our inferences, we do so with candour and a feeling of respect.

---

